# Empowered and engaged: Group exercise for adolescent depression – perspectives from adolescents, parents and healthcare professionals

**DOI:** 10.1177/20503121231225340

**Published:** 2024-02-03

**Authors:** Rebecca Mortazavi, Rebecca Andersson, Håkan Jarbin, Ingrid Larsson

**Affiliations:** 1 Faculty of Medicine, Department of Clinical Sciences, Child and Adolescent Psychiatry, Lund University, Lund, Sweden; 2 Child and Adolescent Psychiatric Clinic, Region Halland, Halmstad, Sweden; 3 Centre for Psychiatry Research, Department of Clinical Neuroscience, Karolinska Institutet, & Stockholm Health Care Services, Region Stockholm, Stockholm, Sweden; 4 Department of Health and Nursing, School of Health and Welfare, Halmstad University, Halmstad, Sweden; 5 Spenshult Research and Development Centre, Halmstad, Sweden; 6 Department of Clinical Sciences, Section of Rheumatology, Lund University, Lund, Sweden

**Keywords:** Adolescents, aerobic, depression, exercise, multiple perspectives, qualitative research

## Abstract

**Objectives:**

Depression is increasing and is a leading cause of disease burden among adolescents. Available evidence-based treatments with medication or psychotherapy have modest effects. Aerobic exercise is a hopeful alternative as an augmenter or a stand-alone treatment. Qualitative studies have shown that participants in group exercise for adolescent depression experienced improved mood and a sense of achievement, commitment and empowerment. This study aimed to explore not only adolescents’ but also parents’ and healthcare professionals’ experiences of a group exercise intervention for adolescents with depression.

**Methods:**

Nine adolescents who had participated in a group aerobic exercise intervention for 12 weeks, eight parents and two healthcare professionals were interviewed. We used a latent qualitative content analysis with an inductive approach that resulted in nine sub-categories, three categories and an overarching theme.

**Results:**

The experiences of a group exercise intervention for adolescents with depression were expressed in the overarching theme ‘Group exercise for adolescent depression promotes empowerment and engagement in everyday life’, based on three categories: exercise alleviates depressive symptoms, exercise contributes to balance in life and exercise promotes self-esteem. However, there was variation in our results, in that not all participants experienced improvements from exercising. Adolescents described more varied experiences, while parents and healthcare professionals mainly expressed positive views.

**Conclusions:**

Our findings suggest that group exercise for adolescent depression promotes empowerment and engagement in everyday life, according to adolescents, and more clearly so according to parents and healthcare professionals.

## Introduction

Depression is a major cause of disease burden among adolescents worldwide.^1^ Epidemiological studies suggest that the 1-year prevalence of adolescent depression is 11%–15% and has been increasing in recent decades.^2,3^ Depression can lead to social and educational impairments,^4^ increased risk of substance misuse, self-harm^5^ and suicide.^4^ In addition, sedentary behaviour and depression in adolescents may increase cardiovascular risk factors and disease.^6^ Loneliness has been correlated to high smartphone access and internet use, and negative affects such as feeling miserable and sad^7^ and may also be associated with depression.^8^

Available evidence-based treatments for adolescent depression, for example, cognitive behavioural therapy, interpersonal therapy^9^ and selective serotonin reuptake inhibitors (SSRIs),^10^ have been shown to have significant but only modest effects. Thus, it is urgent to develop novel treatments for adolescent depression. Aerobic exercise appears to be a feasible and possibly an effective augmenter or option to existing treatments. Meta-analyses on exercise for adolescents with depression, albeit with methodological shortcomings, indicate an effect size similar to psychotherapy and SSRIs.^9–13^

Research exploring how adolescents with depression experience exercise is emerging and may contribute to a better understanding of exercise as a potential treatment for adolescents with depression. Previous qualitative studies have shown that group exercise for depression contributes to commitment and empowerment among adolescents^14^ and that they found group exercise comprehensible, manageable and meaningful.^15^ Furthermore, exercise was described as improving mood and giving a sense of achievement.^16^ A previous study has shown that facilitators for continued exercise included the presence of companionship during exercising, achieving and appreciating exercise results, and the presence of a supportive environment. On the contrary, barriers to maintaining exercise were fatigue, social anxiety, lack of drive and social support.^17^

In this qualitative study, the objective was to widen the perspective and explore the experiences of not only adolescents but also of parents and healthcare professionals (HPs) on how a group exercise intervention affected adolescents with depression.

## Methods

### Design

This study was explorative and we used latent qualitative content analysis with an inductive approach to provide an understanding of adolescents’, parents’ and HPs’ experiences of a group exercise intervention for adolescents with depression.^18,19^ We adhered to the Consolidated criteria for reporting qualitative research (COREQ 32)-item checklist for reporting qualitative research (Supplemental Material 1).^20^

### Setting and intervention

This qualitative study recruited participants from a pilot study conducted prior to a multicentre randomised controlled trial, which was designed to evaluate moderate to vigorous group aerobic exercise compared to group leisure activities for adolescents with a mild to moderate episode of major depressive disorder. The pilot study was conducted in a child and adolescent mental health service (CAMHS) setting in southern Sweden. The adolescents were randomised to group exercise supervised by a personal trainer and supported by HPs or group leisure activities led by the same HPs. The groups met for 1 h three times a week for 12 weeks from March through May 2021. The HPs supported the adolescents with reminders and encouragement before and during the sessions. Each week, there were three different sessions, one session of pure aerobic exercise, one session of strengthening exercise and one mixed session. For the pure aerobic sessions, the intensity at sessions 1–18 was aimed to be at 80%–85% of maximum heart rate for about 21 min, and at sessions 19–36 the aim was 85%–90% for 28 min. Participants allocated to group leisure activities were offered participation in exercise sessions from September to November 2021. For a more detailed description, see the study protocol.^21^

### Participants

**Figure 1.**
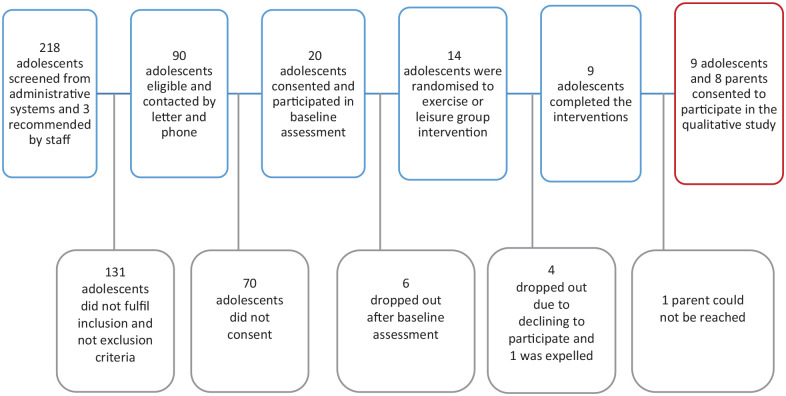
Enrolment in the study. Blue boxes refer to exercise intervention, and red box refers to the qualitative study.

Participants for the exercise intervention were identified from administrative systems but in three cases by recommendation from staff at the CAMHS from November 2020 to January 2021 (Figure 1). Inclusion criteria were adolescents aged 13–17 with major depression, with a current mild to moderate episode, who had attended a minimum of three clinical visits at CAMHS to receive some basic psycho-educational interventions for depression but without a clear response, as assessed through clinical records. Exclusion criteria were eating disorder, high suicide risk, intellectual disability, engaging in regular physical activity of at least 150 min per week at moderate intensity or 75 min per week at high intensity, recent adjustment of antidepressant treatment within the past 4 weeks or stimulants within the past 2 weeks, in need of an interpreter, social circumstances interfering with regular exercise schedule and ongoing psychotherapy.

In total, 14 adolescents were randomised to the interventions and five dropped out; four were due to declining to participate and one was excluded due to immature behaviour during sessions. The nine adolescents who completed the exercise intervention, their parents and the two HPs were invited by phone to qualitative interviews about their experiences. Nine adolescents, eight parents and the two HPs chose to participate, resulting in 19 qualitative interviews (Figure 1). The parent who did not participate in the qualitative study could not be reached. There were eight pairs of adolescents and parents, while one adolescent participated alone. Participants’ demographic and sociodemographic data are presented in Table 1. The adolescents had moderate and close to severe symptoms of depression, measured with Children’s Depression Rating Scale – Revised (CDRS-R), and Clinician-rated Quick Inventory of Depressive Symptomatology – Adolescent Version-17 (QIDS-A17-C). CDRS-R median score was 62 at baseline. It is a 17-item depression rating scale used in research to measure the severity of depression from clinical interviews of adolescents. The total score is 17–113, with a score of ⩾40 indicating depression.^22^ QIDS-A17-C median score was 15. It has a total score ranging from 0 to 27, with a score between 6–10 indicating mild depression, 11–15 indicating moderate depression and 16–20 indicating severe depression.^23^ The adolescents in our study had a moderate to severe functional deficit as well with a median score of 41, measured with the Children’s Global Assessment Scale (CGAS).^24^ CGAS is a clinician measure of global functioning between 0 (lowest) and 100 (highest). The adolescents had a depression duration of between 9 and 72 months (median 36 months), 56% had comorbid attention deficit disorder and 67% had any anxiety disorder, post-traumatic stress disorder or selective mutism indicating a chronic course with extensive comorbidities.

**Table 1.** Demographic and sociodemographic data of participants in the exercise intervention.

Variables	Adolescents (*n* = 9)	Parents (*n* = 8)	HPs (*n* = 2)
Age years (median (range))	16 (13–17)	48.5 (32–55)	45 (37–53)
Sex (*n*)			
Female	6	8	2
Male	3	0	0
CDRS-R baseline^a^ (median score (range))	62 (42–72)	–	–
QIDS-A17-C baseline^b^ (median score (range))	15 (8–23)	–	–
CGAS baseline^c^ (median score (range))	41 (31–55)	–	–
Duration of depression (median months (range))	36 (9–72)	–	–
Co-morbidity with ADD^d^ (percent)	55.6	–	–
Co-morbidity with anxiety disorder, PTSD^e^ or selective mutism (percent)	66.7	–	–
Civic status (*n*)			
Co-habiting	–	4	–
Living alone		4	
Educational level (*n*)			
Lower secondary school	–	0	
Upper secondary school		3	1
University/university college		5	1
Employment (*n*)			
Full time	–	5	2
Part time		1	
Sick leave		2	
Profession (*n*)			
Assistant nurse	–	–	1
Social worker			1
Experience in child and adolescent psychiatry (years)	–	–	15.5 (1–30)

a Children’s depression rating scale – revised. Clinician interviews, total scores 17–113, ⩾40 points indicate depression.

b Clinician-rated quick inventory of depressive symptomatology – adolescent version-17. Total score 0–27, mild depression 6–10 points, moderate depression 11–15 points, severe depression 16–20 points.

c The Children’s Global Assessment Scale, assessing psychiatric functioning on a scale from 1 (lowest) to 100 (highest).

d Attention deficit disorder.

e Post-traumatic stress disorder.

### Data collection

The qualitative interviews were performed by an independent and experienced researcher (IL), and a psychologist (RA) who had no previous connections with the participants. None of the interviewers participated in the exercise intervention or worked at the local CAMHS, and thus had no prior knowledge of the participants. Adolescents, parents and HPs were interviewed separately, except for one adolescent who requested to be interviewed while her mother was present. Adolescents, and one parent (on request), were interviewed by video call, while most parents and HPs were interviewed by phone. Five adolescents randomised to exercise were interviewed after the 12-week intervention in May 2021. Four adolescents, randomly assigned to leisure activities, were involved in exercise sessions either before or after the leisure group. Two were interviewed regarding their exercise experiences directly after the exercise intervention but two had exercised about 6 or 12 months earlier. All parents and HPs were interviewed in November 2021 and thus mostly 5 months after the closing of the exercise session due to a pending ethical approval for extending interviews to parents and HPs. A semi-structured interview guide was used to probed experiences of the exercise intervention. The questions referred to the experiences of the exercise intervention from the adolescents’, parents’ and HPs’ perspectives, with a focus on physical and mental effects. For example, we asked the adolescents, ‘What does exercise mean to you?’ ‘Has exercise led to any changes? How?’ and ‘How do you experience that group exercising affects everyday life?’ The parents and HPs were asked, for example: ‘How do you estimate that group exercise has affected your child’s/the participants’ depression?’ and ‘How do you experience that group exercise has affected your child’s/the participants’ everyday life?’ The interview guide was derived from our previous qualitative study on exercise and depression.^14^ The interview guide can be found in our study protocol.^21^ Two pilot interviews were conducted, and no adjustments were deemed necessary. Therefore, these interviews were included in the study. The interviews varied in duration, ranging from 25 to 112 min, with a median length of 50 min. In total, the interview time was 17 h and 36 min. All interviews were audio-recorded and transcribed verbatim.

### Data analysis

The transcribed interviews were analysed by latent qualitative content analysis.^18^ The transcripts were read through. Parts that were associated with the aim, that is, exploring adolescents’, parents’ and HPs’ experiences of the impact of group exercise on adolescents with depression, were identified as meaning units by RM and RA. We identified a total of 508 meaning units; 211 from adolescents, 228 from parents and 69 from HPs. The meaning units were labelled with codes that shortly described their content. We grouped all codes into nine sub-categories, based on their similarities and differences at a manifest level. The aim was to ensure that each code should not fit in more than one category. The nine sub-categories were subsequently grouped into three main categories, and the latent meaning of the content was abstracted to an overarching theme. For an example of the coding tree, see Table 2. RM and RA discussed the findings several times, and the results were discussed with co-authors until a consensus was reached. The research group was interdisciplinary and included a child and adolescent psychiatrist (HJ, male), a resident in child and adolescent psychiatry (RM, female), a clinical psychologist (RA, female), and a registered nurse (IL, female) with experiences in child and adolescent psychiatric care (RM, HJ and RA) and qualitative methods (IL). RA and IL had no previously established relationship with the participants.

**Table 2.** Example of the coding tree.

Meaning units	Codes	Sub-categories	Categories	Theme
You feel that you could run, or jog, longer if you had to.	Better physical fitness	Physical symptoms	Exercise alleviates depressive symptoms	Group exercise for adolescent depression promotes empowerment and engagement in everyday life
I see it in many of the adolescents, how, at the end of the exercise, when they gather themselves, that there will be a different kind of smile.	Smiles after exercising	Emotional symptoms		
It feels like he/she is more motivated. More motivated for life.	More motivated to life	Cognitive symptoms		
It became a little easier to get out of bed, and things like that, so lots of little things technically.	Easier to get up in the morning	Daily routines	Exercise contributes to balance in life	
Many are much more talkative at the end. They want to connect more.	Talks more	Sociability		
Since he/she felt more energetic, he/she found it easier to go to school.	More energy to school	School performance		
I read a lot before I kind of got depression. . . . And then at the beginning of my depression I also read, but then it kind of disappeared completely. And now I’m trying to pick it up again.	Reengage in hobbies	Leisure activities		
He/she felt satisfied that he/she did it, that he/she did not drop out. Because they had the opportunity to do that. He/she decided to continue.	Satisfied that he/she continued the whole exercise period	Sense of accomplishment	Exercise promotes self-esteem	
I want to exercise more now than I wanted to before.	Motivated to exercise	New attitude to bodily health		

### Ethical considerations

The study received approval from the Swedish Ethical Review Authority (ref. 2020-03364 and 2021-04029). All adolescents, parents and HPs received both oral and written information about the study. Written informed consent was obtained from adolescents and their parents for the adolescents’ participation, as well as from the parents who were interviewed, and HPs. Participants were free to withdraw from the study at any point.

## Results

Adolescents, parents and HPs experienced that a group exercise intervention for adolescents with depression promoted empowerment and engagement in everyday life. Exercise promoted empowerment and engagement in that exercise was experienced to alleviate depressive symptoms, contribute to balance in life and promote self-esteem. Changes in emotional symptoms with increased positivity, impact on daily routines, sociability and a sense of accomplishment are examples of how exercise contributed to the adolescents’ empowerment and engagement (Table 3). Exercise also led to a new attitude to bodily health regarding the ability to affect one’s health, and thus strengthened the adolescents’ empowerment. However, the results varied somewhat and not all participants experienced improvements. The categories and sub-categories were illustrated by quotations from adolescents, parents, and HPs (Table 4).

**Table 3.** Overarching theme, categories and sub-categories exploring adolescents’, parents’ and HPs’ experiences of the effects of a group exercise intervention on adolescents with depression.

Overarching theme	Group exercise for adolescent depression promotes empowerment and engagement in everyday life		
Categories	Exercise alleviates depressive symptoms	Exercise contributes to balance in life	Exercise promotes self-esteem
Sub-categories	Physical symptoms	Daily routines	Sense of accomplishment
	Emotional symptoms	Sociability	New attitude to bodily health
	Cognitive symptoms	School performance	
		Leisure activities	

**Table 4.** Quotations from participants that illustrate their experiences of the group exercise.

Categories	Sub-categories	Adolescents’ quotations	Parents’ quotations	HPs’ quotations
Exercise alleviates depressive symptoms	Physical symptoms	*It was probably my energy level, which I noticed first. That it started to increase, I had more stamina and I wanted to be more physically active. That was probably the first thing to change. (A6)*	*My adolescent felt more alert, could handle people and had more energy than before, so that also affected the mood. (P-A6)*	*I think they had pretty high confidence in this trainer, who could say ‘this kind of pain is exercise soreness and nothing dangerous, so you can continue exercising’. You noticed that some had never felt it before and found it a bit scary. (HP2)*
	Emotional symptoms	*I didn’t feel like the exercise made me feel any better. The only thing it did was make me more stressed. Because it was so early after school. I had to hurry away from school, leave early sometimes.* (*A5)*	*My adolescent felt a lot better for a little while after [exercising]. . . . and says it was the only period in many, many years feeling reasonably well. (P-A8)*	*In a way, they are a little freed up from their usual worries and thoughts because they have to stay focused on the instructions. (HP1)*
	Cognitive symptoms	*Now, I have more hopes, dreams and ideas about the future and stuff like that. I have more small goals along the way and things that make me more motivated. It doesn’t feel like I’m just treading water for several days. (A6)*	*It won’t always be like this. You can have a future. You don’t have to wait until you’re an adult. Even as a youth you can have a future. I think that is important. (P-A9)*	*–*
Exercise contributes to balance in life	Daily routines	*It is like a trinity with sleep, food and exercise, I think. It is important to keep them in check and perhaps to be social. (A3)*	*On the one hand, it [exercise] is good for the body, you sleep better, you may eat a little better, you get more rhythm throughout the day. (P-A2)*	*If you come to the training, all of a sudden it is not as difficult to go somewhere else. Maybe to see friends a little more. It feels like they get some form of increased harmony and balance, but it’s a feeling from the outside when you see them in the room. They look calm, safe compared to how they looked in the beginning. (HP1)*
	Sociability	*It [the exercise group] meant that I quite often got to be around people I didn’t know very well, and it made it easier for me to be around people. (A1)*	*It has become much easier to, for example, have other relatives visiting the family. My adolescent takes part and feels appreciated and does not leave as soon as possible after dinner, for example. (P-A1)*	*For me, I feel that this was much more for these teenagers than just physical exercise to reduce their depression. It was a new social context that might become a positive experience that some of them take with them. (HP2)*
	School performance	*Many days when I had the exercise, I also went to school, because it was kind of easier to get to the exercise on the days I went to school too. Then I got a better structure at school. (A6)*	*My adolescent still dared to go [to school] for a while last fall and managed to join a group. I think that it has a lot to do with participating in a group setting while exercising. My adolescent had gained a positive experience. (P-A4)*	*Some [adolescents] have been at school much more and have dared to participate in sports at school. (HP1)*
	Leisure activities	*I have been trying to pick up hobbies that I lost interest in when I was very depressed. . . . It’s kind of working. (A6)*	*My adolescent has become more interested in spending time outside home and dares to do a little more. (P-A4)*	*They [the adolescents] have described that they have started to exercise a bit, for example been out jogging with mum or dad. (HP1)*
Exercise promotes self-esteem	Sense of accomplishment	*Before I started exercising, I ran my usual lap, which is maybe 4* *km, in maybe 30* *min and now I’m down to, like, 22. It’s something that makes you, like, wow, now I can do this or now I can do this too. (A3)*	*I think my adolescent understands that it [the group exercise] builds confidence and makes you feel good. To feel stronger as well. A realisation that it is good for you. (P-A5)*	*It is also very empowering to be someone who manages to participate in an activity. To show up even when it was difficult. It was a big challenge on so many levels. (HP2)*
	New attitude to bodily health	*It [exercise] is fun. It’s nice afterwards, when you’re done, to feel that you’ve done something good for your body. (A2)*	*My adolescent actually developed a need to exercise and discovered this as a way to cope, to feel better. (P-A3)*	–

A: adolescent; P: parent; HP: healthcare professional.

### Exercise alleviates depressive symptoms

Exercise alleviated depressive symptoms through changes in physical and emotional symptoms, along with a beneficial impact on cognitive symptoms.

#### Physical symptoms

Group exercise led to improvements in physical vigour, such as increased energy levels and becoming physically stronger, according to adolescents, parents and HPs. The increased energy levels indicate an alleviation of depressive symptoms. According to HPs, some adolescents experienced sensations in their bodies that were entirely new to them, such as being sore after exercising or feeling their heart beating during exercise. Adolescents could also describe how exercise improved their body posture and alleviated previous problems with neck and back pain. Some adolescents and parents did not describe improvements in physical symptoms. Instead, they experienced more tiredness because they had spent all their energy on the group exercise, which meant that it was difficult to take part in other activities after an exercise session or during the 3-month intervention.

#### Emotional symptoms

Adolescents described that they were generally happier and less irritable, which indicates a reduction in core depressive symptoms. One adolescent described that the increased positivity influenced people in their surroundings, leading to a better atmosphere. Some adolescents described that, during exercise, they were temporarily distracted from worries. By contrast, some adolescents did not experience improved well-being, and some described that they were more depressed, with one describing having more suicidal thoughts. Some adolescents were stressed about leaving school early to make it to the exercise sessions. Parents mainly described their adolescents as being more positive, happier and less irritable and thought this was due to the group exercise. However, one parent described increased stress due to the exercise schedule and connected difficulty in coping with life to suicidal behaviour. HPs noticed adolescents smiling after exercise sessions, seemingly less irritable and angry. They also described that, while the adolescents were focusing on the exercises, they had less time to focus on worries or dark thoughts.

#### Cognitive symptoms

Adolescents and parents, but not HPs, described that exercise alleviated cognitive symptoms of depression. Adolescents reported improved memory, less negative thinking and being more oriented towards the future. Parents described better concentration among adolescents and that they were more motivated to live and had hope for the future.

### Exercise contributes to balance in life

Exercise contributed to balance in life with impact on daily routines, such as sleep, eating and rhythm during the day, sociability, school performance and involvement in leisure activities.

#### Daily routines

Some adolescents experienced a difference in their daily routines in the form of better eating, improved sleep and how exercise created a healthy routine. Others described more difficulties falling asleep or no change in sleeping patterns. Adolescents also described that exercise became something to fill their day with, and regular exercise gave structure to everyday life. Parents mostly described a positive impact on daily routines from exercise sessions, noticing better sleep, eating habits and a better balance in life. According to HPs, adolescents gained a better everyday structure from an exercise schedule.

#### Sociability

Adolescents found it easier to be around people. They felt supported by the group in the exercise sessions and spent more time with family and friends. This increased ability to participate in social settings contributed to a more balanced daily life. However, some adolescents experienced no change in sociability. Parents described their children as being more outgoing, less isolated and talking more. They noticed fewer conflicts at home. Parents could also describe how the adolescents got support in the group and gained a sense of togetherness. HPs noticed the adolescents being supportive of each other during exercise sessions. They described how the adolescents started chatting more and some also seemed to have a better relationship with their parents. Some adolescents did also seek support and shared feelings of disappointment with HPs if they were not feeling better after the exercise intervention.

#### School performance

Adolescents described both improved performance and attendance at school. Parents described that their adolescents had more energy for school. One parent noticed that the adolescent dared to go back to school after working out with peers at exercise sessions. However, some adolescents and parents saw no change or even a negative effect on school, for example, due to less school attendance. HPs described that adolescents reported better school attendance in some cases but also that some adolescents and parents were worried about adolescents missing school due to exercise sessions. Since school is a major part of adolescents’ lives, participation in school promotes balance in life.

#### Leisure activities

Adolescents experienced that group exercise had an impact on their leisure activities. They became more active, and again enjoyed their old hobbies, contributing to a better balance in life. Parents described that the adolescents became more comfortable with people. They managed to go shopping or take a walk with the family and were more interested in having a leisure time outside the home. HPs described that adolescents reported engagement in sports in their spare time and found it easier to do other things at home, for example, going out for a walk.

### Exercise promotes self-esteem

This category describes how participants experienced that exercise promoted self-esteem, through a sense of accomplishment and a new attitude to bodily health.

#### Sense of accomplishment

Adolescents described that they felt proud of themselves because they took part in exercising. Managing exercise gave them confidence in their own abilities. They described feeling more secure with themselves, and some also experienced improved self-acceptance. However, some adolescents experienced no change in self-confidence. Parents saw their children being satisfied with themselves and proud after the workout sessions. Parents described that exercise boosted adolescents’ self-confidence when they got stronger and performed something beneficial for their health. According to parents, their adolescent had matured from the exercise intervention. HPs gave several specific examples of how they noticed improved self-esteem among adolescents. For instance, they could enter the gym without their parents’ support, dared to laugh and show their feelings and were even a bit mischievous. They dared to chat, approach the coach and ask questions once they had participated in the intervention for some weeks. In the beginning, girls put make-up on before going to the gym but they were not doing so by the end of the intervention.

#### New attitude to bodily health

Adolescents and parents, but not the HPs, described how the exercise intervention contributed to a new attitude to bodily health. Adolescents reported more motivation to exercise, they found it more enjoyable and saw exercise as a way of taking care of the body. Parents noticed their children taking more initiative to exercise, that they found exercising important and expressed a need to exercise. The new attitude to bodily health strengthened the adolescents’ confidence in being able to improve their own health, which also promoted their self-esteem.

## Discussion

This qualitative study, with experiences not only from adolescents but also from their parents and HPs, elicited experiences of group exercise that range from impact on depression symptoms, and balance in life, to self-esteem, thereby promoting empowerment and engagement in everyday life. However, there is variation in our results, and not all participants experienced a beneficial effect or empowerment from exercising. Adding parental and HP perspectives showed changes in line with those reported by the adolescents, albeit the parents’ and HPs’ views were generally more positive than those of the adolescents. Although adolescents, parents and HPs report beneficial effects of exercise on depression, exercise is not yet, with robust methodology, proven to be effective and, as of today, certainly not a replacement for other evidence-based treatments such as psychotherapy and SSRI.

Our results are supported by previous findings exploring adolescents’ experiences of exercise interventions. Group exercise interventions for adolescents with depression have shown reduced depressive symptoms and improved well-being,^14–17^ structure in everyday life and routines,^14–16^ as well as an effect on achievement and self-esteem.^14–17^ How exercise promotes empowerment and engagement in everyday life became clearer with the addition of parents’ and HPs’ perspectives in our study, confirming earlier findings with multiple perspectives. In our study, adolescents expressed a broader spectrum of experiences from the exercise intervention, while parents and also HPs described more beneficial aspects of routines, and how the adolescents became more outgoing, sociable and self-confident. Informant discrepancies are a well-known phenomenon in assessing adolescent depression, where parents and adolescents often give divergent reports of symptoms.^25^ There is less correspondence between parents and adolescents for internal symptoms that are not externally visible, such as depression and anxiety, than for external symptoms.^26^ This can explain why, in our current study, adolescents had more varied experiences of the exercise intervention compared to parents’ and HPs’ mainly positive views. We looked through existing heart rate data from exercise sessions but could not find any correlation between the adolescents’ number of minutes above 80% of maximum heart rate and how the adolescents experienced the intervention. HPs described no cognitive changes or new attitudes to bodily health, possibly due to thought content not being shared in an exercise session.

This study shows that exercise may promote empowerment and engagement in everyday life among adolescents. Empowerment is a construct that involves perceptions of personal control and a proactive approach to life.^27^ In our study, we found that exercise increased empowerment and engagement, in terms of experiences of improved mood, more balance in life, improved self-esteem and motivation to maintain exercise after the intervention. The empowerment from exercise, described by the participants in our study, may resemble the prevention of relapse in cognitive behavioural therapy, for example, described in Kennard’s 2008 study.^28^ Thus, the increased empowerment and engagement in everyday life for adolescents in our study might possibly prevent relapse by better managing life challenges.

The results revealed that exercise contributed to better daily routines and balance in life, by following a regular exercise schedule. Although exercise is not a proposed mediator of behavioural activation therapy, establishing a regular exercise routine is often an important part of the therapy, which has been shown to be effective for adults with depression.^29^ Behavioural activation therapy has shown promising results for adolescents,^30,31^ and has been found to be preferred among young people to cope with depression.^32^ Development of healthy habits among children is described as important to their health in the long term.^33^ Exercising regularly may be a way of establishing healthy habits and of not only reducing negative emotions but also increasing positive emotions and balance in life.

The group setting, and not specifically the exercise, may improve sociability and thus counteract feelings of loneliness and negative emotions in adolescents. This design cannot address whether the group setting or the exercise contributes the most to increased sociability. Parents and HPs described a better family climate and less conflicts at home. This could be driven by the immediate relief from anxiety or negative emotions after each session and sharing this positive moment with parents on the way back home.

Even though adolescents, parents and HPs in our current study experienced exercise as empowering, through improved mood, better balance in life and improved self-esteem, not all participants described these effects. Exercising could be beneficial for adolescent depression but does not apply to everyone. Exercising regularly may also be perceived as a demanding treatment, which requires motivation from the patient. Some adolescents experienced the exercise intervention as stressful and intrusive on their school schedule. This stress can be mitigated by scheduling exercise sessions at later hours and on weekends or reducing the workload in school to allow for exercise for this affected group of adolescents.

A first step for future research is to provide robust evidence on the effects of exercise on adolescent depression. If proven to be effective, future studies should address when group exercise could be provided as a stand-alone treatment or as an add-on to other evidence-based treatments, for which subgroups, and when, in the course of treatment, exercise is most beneficial. For future qualitative research, conducting studies with participants recruited from multiple sites and lower tiers of care with less chronic and less co-morbid participants would be interesting. Another focus of qualitative studies is to explore the experiences of participants who did not complete the intervention in order to adapt the intervention to different needs.

### Strengths and limitations

The concepts of credibility, dependability, confirmability, transferability and authenticity are used to describe various aspects of trustworthiness in qualitative research.^18,19^ Credibility was strengthened by using data from adolescents, parents and HPs with 19 individual interviews, resulting in a sufficient number of meaning units to detect similarities and differences, approaching saturation. By adding interviews with parents and HPs, new perspectives were provided. Despite the COVID-19 pandemic in 2020, this study was still feasible in Sweden at that time, given that there was no lockdown and primary schools were still open, while secondary schools had distance-based education for a limited time.^34^ Although the pilot study had a small number of participants due to the pandemic, we reached enough participants for this qualitative study and achieved variation regarding variables such as age, gender and severity of depression symptoms. Malterud et al.^35^ describe the concept of ‘information power’ used to evaluate sample size in qualitative studies. They state that the more information the sample in a study holds, the lower the number of participants needed to reach sufficient information power. The sample size can thus be lower for a study with a specific aim, specificity of participants’ experiences, quality of interview dialogue and use of explorative analysis. Thus, this study satisfied these criteria for allowing this small but sufficient sample size. The interviews were coded by researchers RM and RA, who discussed the findings several times, and the results were furthermore discussed with co-authors until a consensus was reached. Examples with representative quotations from the transcribed text were presented to increase credibility. Dependability was enhanced by interviewing most participants individually, using the same interview guide with open questions, followed by encouragement to speak openly. Using video and phone interviews could represent a limitation when creating contact compared to face-to-face interviews. Another limitation was that all interviews with parents and HPs and a few with adolescents were conducted at least 6 months after the intervention. This may have affected their memories from the intervention, and possibly amplified positivity from parents as minor inconveniences tend to be forgotten with time. The interviews were performed by an independent and experienced researcher (IL), and a psychologist (RA). Using two interviewers can lead to variation in interviews and could be considered as a weakness but it also represents a strength, as more varied perspectives can be elicited. Given that the interviewers did not work at the local CAMHS and had no previous relation to the participants, the probability that the participants were able to openly share their experiences is increased. Confirmability was strengthened as all authors had different backgrounds in the fields of health and medicine with a range of experiences, which resulted in an increased ability to understand the participants’ experiences. A limitation was that the participants did not have the opportunity to read the transcripts from their interviews to correct potential misunderstandings. Furthermore, the population was recruited from one local CAMHS, with rather low sociodemographic variation, which could be considered as a limitation in terms of transferability. Our population had moderate and close to severe depression ratings and loss of function and cannot directly be translated to primary care populations, given the degree of difficulty. However, the overall agreement regarding the identified categories between adolescents, parents and HPs increases the transferability of our results. Authenticity was strengthened by the variability in participants’ experiences and the varied perspectives among adolescents, parents and HPs.

## Conclusion

Group exercise for adolescent depression may promote empowerment and engagement in everyday life, by alleviating depressive symptoms, contributing to balance in life and promoting self-esteem. Our findings suggest that group exercise has benefits among some adolescents with depression according to themselves, and more clearly so according to parents and HPs.

## Supplemental Material

sj-docx-1-smo-10.1177_20503121231225340 – Supplemental material for Empowered and engaged: Group exercise for adolescent depression – perspectives from adolescents, parents and healthcare professionals Supplemental material, sj-docx-1-smo-10.1177_20503121231225340 for Empowered and engaged: Group exercise for adolescent depression – perspectives from adolescents, parents and healthcare professionals by Rebecca Mortazavi, Rebecca Andersson, Håkan Jarbin and Ingrid Larsson in SAGE Open Medicine
